# Salicylic Acid

**Published:** 1876-10

**Authors:** Daniel T. Nelson

**Affiliations:** Prof. Physiology and Histology, Chicago Medical College; Chicago, 1108 Indiana Avenue


### SALICYLIC ACID.

By DANIEL T. NELSON, M.D.,

Prof. Physiology and Histology, Chicago Medical College.

The following cases, except the last, were treated in the Mercy Hospital, and the report is made from the carefully kept records of Dr. Fred. C. Schaefer, the interne in the medical wards.

Case I. *Intermittent Fever.* H. E., a locksmith, male, German, single; admitted to hospital May 4, 1876, 4 p. m., discharged May 18, 1876. Was taken sick in Memphis, Tenn., about two weeks ago with chills and fever. Paroxysms recur every second day; last at 5 p. m. yesterday. Temperature 99°, pulse 80, skin dry, urine scanty and dark colored, lips covered with fever sores, (herpes,) tongue heavily coated, yellowish-brown color, appetite poor, thirsty, some cough. Had a dose of magnesia sulphate on entering. Bowels moved freely at 10

P. M. Acid salicylic ℥j. in gr. x. doses ordered to be taken daily between paroxysms.

May 5th, 8 A. M. Pulse 72, temperature 99°, resp. 22. Has headache in frontal region, coughs considerably, bowels moved this morning, no appetite, sweat freely last night. Had a chill at 5 P. M., lasting 1½ hours, followed by high fever.

May 6th, 8 A. M. Had a dark-colored stool this morning, tongue still coated, head aches, pulse 92, skin moist, temperature 98½°, has ringing in the ears. Ordered acid salicylic gr. xv. four times a day. Noon—pulse 84, temperature 98½°, appetite returning, stools yellowish.

May 7th, 8 A. M. Pulse 84, full and moderately firm, temperature 98½°. 4:30 P. M.—having a chill; temperature during chill, 101°, in axilla. Give acid salicylic gr. x. every hour till bedtime after " fever " returns.

May 8th, A. M. Much better, pulse 84, temperature 98½°, appetite good, feels a little " sore " in stomach. Ordered acid salicylic gr. x. every two hours to-day.

May 9th. Is walking about; "feels good;" acid salicylic gr. x. three times to-day.

May 10th. Still improving; pulse 4 P. M., sitting, 65.

May 11th. Convalescent; acid salicylic gr. x. three times to-day.

May 12th. Took acid twice to-day.

May 13th. Had a chill 10:30 A. M., lasting half an hour. Has pain in knees and elbow joints. Ordered acid salicylic gr. x. every three hours.

May 14th. Better.

May 15th. Had a chill 10 A. M., lasting nearly half an hour. The supply of salicylic being exhausted, quiniæ sulph. gr. v. was given every four hours.

May 16th. Resumed salicylic acid, half drachm daily. Pulse 96; appetite fair; rested better last night; stools yellowish.

May 17th. Slept poorly last night; dizzy all the time; appetite good; walks about the ward; pulse 72; temperature 98½°; takes acid salicylic gr. x. three times to-day.

May 18th. Convalescent; discharged. Visited hospital May

20th; no return of chills; has regained strength. Took during illness ℨix. of salicylic acid, and gr. xv. of quinine.

Case II. *Intermittent Fever.* T. M., admitted May 23, 1876, 6 P. M., discharged May 29, 1876. Male; age 35; boiler maker. Has been unwell some nine weeks; had an attack of "chills and fever," lasting some five weeks at beginning of illness. Chill came every two to four days; got some better. Chills returned ten days ago. Has had a chill every evening for a week; chill lasts from half to two hours; last chill yesterday, 8 P. M. Temperature 99½°, pulse 98, respiration 20, urine abundant but dark in color; tongue dry in center, brown coat on either side; appetite poor; thirst great. *Treatment.*—6 P. M., acid salicylic gr. x. every hour till bedtime.

May 24th, 8:45 A. M. Pulse 84, moderately full; respiration 20, temperature 98½°. Took 30 grs. last night before bedtime. Had no chill; slept well; is much refreshed. Bowels moved before breakfast; did not observe color of stool; appetite improving.

May 25th, 8:30 A. M. Pulse 84, respiration 20, temperature 98½°. Had no chill last night. Took five ten grain doses during the day, yesterday; last dose 5 P. M. Tongue clearing; appetite improving; skin natural; urine normal. Continue acid, five doses of ten grains.

May 26th, 9 A. M. Pulse 88, respiration 22, temperature 98½°. Tongue a little coated; skin natural; no chill; took 50 grains yesterday. Continue acid, ten grains three times a day.

May 27th. Convalescent. Took four drachms acid.

Case III. *Intermittent Fever, with Diphtheria.* A. W., admitted May 2, 1876, age 20; female. Taken sick with sore throat a week ago; slight cough; has a chill twice a day. Temperature 104°, pulse 120; skin dry and hot; tongue brown coat; tonsils much inflamed; opens the mouth with great difficulty. Had quiniæ sulph. gr. iv. every four hours, and for an external application a solution of plumb. acet.

May 3d, A. M. Neck still much swollen, and throat very sore, but opens mouth more easily. Took hydrarg. chld. mit. gr. iij. with bismuth. subnit. gr. ij. last night. Bowels moved freely; stools dark brown; had a chill this morning, 5:30 A. M.

P. M. Ordered acid salicylic gr. x. every hour, to use ℨj. daily.

May 4th, A. M. Is much improved; temperature 102°; pulse 96; had a chill last night at 10 P. M. Continue acid. P. M. Temperature 101°, pulse 92; throat much better.

May 5th, A. M. Temperature 101°, pulse 96; throat still sore; appetite improving; had a chill at 10 P. M. last night. Continue acid ℨj. daily.

May 6th, 8 A. M. Slept well last night; had prolonged feeling of coldness this morning, lasting two hours; skin now warm and moist; temperature 99°; pulse 84; respiration 22; soreness in throat diminishing; has some ringing in ears. To take ℨss. acid to-day. 3 P. M. Had a "chill" for half an hour; has no ringing in ears; temperature 99½°; knees cold.

May 7th, 8 A. M. Improving; walks about the ward.

May 8th. Convalescent; left. Has taken ℨiv. salicylic acid during illness.

May 14th. Returned; throat much swollen again; very painful; had on entering a gargle of potass. chlorat, tr. belladonæ and acid. tannic., which gave some relief.

May 15th, 8 A. M. Considerable inflammation in fauces, with small diphtheritic patches on tonsils; has great pain in right ear. Prescribed cinchonidiæ sulph. gr. v. three times a day; continue gargle.

May 16th, 8 A. M. Awake all night with pain in ear; continue local treatment; apply solution of morphia to ear; acid. salicylic. ℨj. daily.

May 17th, 8 A. M. Earache gone; throat better. 3 P. M. Is feeling much better; can swallow more easily; diphtheritic patches gone.

May 18th, A. M. Very much better; slept better; take gr. x. acid every three hours.

May 19th, 5 P. M. Feeling very cold, though a very warm day; has a shawl closely wrapped about neck and shoulders. 11:30 P. M. In a cold, collapsed state; body covered with a cold perspiration; knees and elbows *very* cold. Ordered ammoniæ carb. internally for stimulant.

May 20th, 11 A. M. Extremities feel very cold to patient; temperature, popliteal $96\frac{2}{5}°$, axillary $99\frac{3}{10}°$; patellæ cold; hands cool; pulse 84, good volume.

May 21st, A. M. Sitting up; takes two doses, ten grains each, of acid.

May 22d. Convalescent; has taken ʒ ijss. acid since relapse.

CASE IV. *Spanœmia, with Neuralgia.* B. G., admitted April 7th, discharged May 8th, 1876; aged 20; female; house maid. Has had backache and headache for about a week; bowels constipated; menses absent five months; has had menses but three times a year for four years; leucorrhœa at times, not now. No dysmenorrhœa; nervous; temperature 100°; pulse 84, full; respiration 18; tongue considerably coated; stomach irritable; has a mitral murmur, probably not organic. *Treatment.*—At first a mixture containing carbolic acid., tr. gelsem, and tr. opii. camph. was used for the irritable stomach, and pills of cinchonidia gr. ij. each, with tr. iron and nitro-mur. acid in mixture for the spanaemia. Then when severe neuralgic pains came on, chiefly in the supraorbital and otic branches of the fifth, recurring periodically though irregularly, cinchonidia in five grain doses was given in the interval of the pain (15 grs. in 24 hours). Temporary improvement. The neuralgia returning, and severe, quinine, gr. 15 in 24 hours in the intervals of the pain was tried. Better results, but still the pain returns, and at times as severe as ever.

May 4th, A. M. Pains very severe; ordered acid. salicylic. gr. x. every two hours.

May 5th, A. M. Pains return only occasionally, and not as severe; pulse 84, temperature $98\frac{1}{2}°$.

May 6th, A. M. Pulse 78, respiration 22, temperature $98\frac{1}{2}°$, bowels natural, appetite good. Acid, gr. x. every three hours.

May 7th. Pain returned at 4 P. M. Take acid gr. x. every two hours.

May 8th. Is much better. Left the hospital with a prescription for salicylic acid, and the injunction to continue it. Took $3\frac{1}{2}$ drachms of the acid. The patient left the hospital sooner than we could have wished, but the acid certainly controlled the pains better than any of the remedies used, so long as she was under observation.

Case V. *Acute Rheumatism with Syphilis.*

Admitted April 12th, discharged May 8th, 1876. F. L. A., age 29; female. During past four weeks has had pain in knees, ankles, shoulders, elbow and wrist, gradually increasing in intensity. Knees and ankles somewhat swollen. Sweats considerably towards night. Had a mixture containing potass. citrat. gr. xl. every three hours until April 14th, P. M., when there being little change for the better, acid. salicylic. gr. vijss. every four hours was ordered.

April 15th, A. M. Slept well last night and is feeling much better this morning; has no pain; joints sore on moving; temperature 98½°, pulse 76, urine alkaline.

April 16th, A. M. Joints more painful; tongue a little coated; appetite poor. Noticed nodes on each tibia, which are quite red and tender. All the joints were painful last night. Has sore throat.

April 17th, A. M. Slept poorly from pain. Continue acid.

April 18th, A. M. Slept well last night; pains slight; joints stiff.

April 19th, A. M. Pains gone entirely; slept well last night.

April 21st. Sitting up; pain returned in knee last night; not severe. Ordered potass. iod. gr. xxx. daily. Continue salicylic. acid.

April 27th. Acid stopped; iodide continued; pain and stiffness all gone; took ʒ vj. acid in all.

Case VI. *Acute Rheumatism.*

M. M., age 25; female. April 22, 1876, took cold by sitting near an open window while perspiring freely. Second day afterward took to bed with severe pains in back and feet; feet swollen; was in bed a week; treated by a physician, but treatment not known. Sat up four days; pains returned and took to bed again.

May 9th. Ordered acid. salicylic. gr. x. every hour till pain relieved. Next day pain all gone; joints sore and stiff. Powders did not disturb the stomach; took them in paper. Continued the powders at longer intervals during the week.

May 21st. Pains returned again, but not so severe. Took powder again every hour till relieved.

May 28th. Has continued the powders at increasing intervals. Appetite has increased constantly while taking the powders. The swelling, soreness, and stiffness gone from all the joints. Has taken in all one ounce of the acid.

Remarks.—The first four cases seem to show that salicylic acid is efficient in controlling periodic fever and neuralgia as well as quinine; indeed, in the one case of neuralgia reported better. The dose required seems to be about four or five times greater than quinine; but the cost of salicylic acid is now less than one-sixth that of quinine, 35 cents and $2.35 per ounce. So the cost of a drachm of salicylic acid would be equal to nine grains of quinine, which would certainly be a small allowance per day for a severe case of intermittent fever or neuralgia, while a drachm of salicylic acid would in most cases be an ample allowance. As with quinine its taste is very disagreeable. But it may be administered in wafers or paper and be tasteless. The dry powder is usually quite readily taken also in any thick syrup. It was given in the hospital, in above cases, in solution as follows:

Acid. Salicylic. .......................... ℥j.
Alcoholis .......................... ℥ vij.

Dissolve and then add—

Ext. Malt } *aa* .......................... ℥ vij.

Aquæ }

Syr. Glycyrrhizæ .......................... ℥ iij.

Half an ounce of this mixture contains ten grains of the acid—the usual dose. If borax in equal quantities with the acid or less, be added to the mixture, the alcohol may be diminished, as the borax renders the acid more readily soluble, though it makes the mixture more liable to disturb the bowels. The acetates of potassa, soda and ammonia also render the acid much more soluble, so that the alcohol may be omitted in the menstruum. The pure acid is more readily soluble than the impure.

Case III. illustrates very well the effects of a full dose—yes, over dose—of the drug.

By the kindness of Drs. Schaefer, Palmer and Sienank, at the Mercy Hospital, I am able to give the following results of experiments made upon themselves to determine the effects of salicylic acid when taken in health. The real effect upon the pulse, respiration, and temperature, is doubtless the most accurately shown in Experiment I., as the Doctor lay quiet during the first three hours of the experiment, while the others were moving about as their duties called them. Yet it will be seen that the results noted substantially agree.

### Experiment I.

Amount taken, gr. xv. in solution at one dose. Weight of man 140 lbs. Normal pulse, (position reclining,) 72. Axillary temperature 98°. Respirations 16. Remained in reclining position three hours. After taking the drug the following observations were made:

| Time. | Pulse. | Resp. | Temp. | |
|---|---|---|---|---|
| 5 Min. | 72 mod. size. | 16 | 98° | |
| 10 | 82 a little bounding. | 18 | 98 1-10° | |
| 15 | 72 full volume. | 13 | 98¼° | |
| 20 | 72 " " | 12 | 98 6-10° | Surface temperature depressed, knees, ankles and elbows cool to the touch. |
| 30 | 72 " " | 11 | 98 4-5° | Surface temperature depressed, knees, ankles and elbows cool to the touch. |
| 35 | 72 " " | 10 | 99° | Limbs cold from knees to middle third of thigh; and from elbows to middle of humerus. Considerable diaphoresis about the joints mentioned. |
| 40 | 72 " " | 9 | 99° | Limbs cold from knees to middle third of thigh; and from elbows to middle of humerus. Considerable diaphoresis about the joints mentioned. |
| 45 | 74 " " | 10 | 98½° | Skin perspiring all over. Joints quite cold to the senses. Head feels "light." Brow very moist. |
| 55 | 74 " " | 13 | 97½° | Knees, elbows and ankles very cold and clammy. Desire to urinate quite marked.* Entire surface of body moist and cool. |

* The bladder was evacuated just before the acid was taken.

| Time. | Pulse. | Resp. | Temp. | |
|---|---|---|---|---|
| 1 Hour. | 74 full volume. | 13 | 98½° | Whole surface of body quite moist but comfortably warm; face a little red; passed half pint of urine.* |
| 1⅓ | 74 " " | 14 | 98½° | Skin *wet* all over. Knees and elbows still cold, while surface temperature is higher than normal. Head feels "full." Feel buoyant. Face flushed. |
| 1⅔ | 80 very full. | 16 | 99° | Feel a glow of heat all over body except the joints, (knees and elbows.) Skin still moist; face very red and warm. |
| 2 | 80 " " | 18 | 98 9-10° | Feel a glow of heat all over body except the joints, (knees and elbows.) Skin still moist; face very red and warm. |
| 2½ | 72 moderate vol'm. | 16 | 98° | Surface temperature, little above normal. Still somewhat moist. Face continues flushed. Passed urine, half pint, light colored.* |
| 3 | 72 " " | 16 | 98° | Surface temperature again natural. Skin only slightly moist. |
| 4 | 72 | 16 | 98° | Only signs present indicating the action of the drug, *cold joints.* |
| 6 | 72 | 16 | 98° | Only signs present indicating the action of the drug, *cold joints.* |

### Experiment II.

Amount taken, gr. x., one dose in pills. Weight of man 150 lbs. Normal pulse 96, sitting. Axillary temperature 98 3-5°. Respiration 18. Urine tested with Tinct. Ferri Chld. After taking the drug the following observations were made. (Pulse and respiration taken in sitting posture.)

| Time. | Pulse. | Resp. | Temp. | | Urine Test. |
|---|---|---|---|---|---|
| 5 M.aft'r. | 100 | 18 | 98 3-5° | | No trace present. |
| 15 | 104 full. | 24 | 98¼° | | Precipitate slight; violet tint. |
| 30 | 96 " | 24 | 98 1-5° | | Precipitate slight; violet tint. |
| 45 | 96 " | 24 | 98° | | Precipitate dark wine color. |
| 60 | 96 " | 24 | 98 2-5° | Skin dry and cool. | Purple color. |
| 2½Hours. | 96 " | 24 | 98 3-5° | Skin moist and cool; knees and elbows cold. | Purple color. |
| 8 | 96 mod. | 20 | 98 3-5° | Skin normal. | Purple color. |
| 50 | 96 | 18 | 98 3-5° | | Wine color. |

* The bladder was evacuated just before the acid was taken.

### Experiment III.

Amount taken, gr. xv., in solution. Weight of man 140 lbs. Normal pulse, sitting, 76. Axillary temperature 97 2-5°. Respiration 16.

| Time. Minutes. | Pulse. | Resp. | Temp. | Urine Test. | |
|---|---|---|---|---|---|
| 4 | 80 | 18 | 97 2-5° | Light violet tint. | |
| 10 | 80 | 19 | | " " " | |
| 20 | 72 | 19 | | | Skin a little moist all over. |
| 30 | 72 | 14 | 96 2-5° | Wine color. | Skin very moist; knees and elbows quite cool. |
| 60 | 72 | 18 | 97 2-5° | | Surface temp. below normal; knees and elbows cold and clammy; entire surface covered with perspiration. |
| 90 | 72 | 21 | 98° | Purplish. | Face flushed and warm; head feels a little light. |
| Hours. | | | | | |
| 2¾ | 72 | 16 | 96¾° | Purple. | Face flushed and warm; head feels a little light. |
| 6 | 72 | 16 | | " | Surface of body normal. |
| 8 | 76 | 13 | 97¼° | " | " " " |
| 48 | 76 | 16 | 97 2-5° | " | " " " |
| 72 | 76 | 16 | 97 2-5° | Urine gives a very faint violet tint. | |

### Experiment IV.

Amount taken, grs. xx., in solution. Weight of man 145 lbs. Normal pulse, sitting, 65. Axillary temperature 98 3-5°. Respiration 16.

| Time. Minutes. | Pulse. | Resp. | Temp. | Urine Test. | |
|---|---|---|---|---|---|
| 5 | 60 strong. | 18 | 98 3-5° | No trace. | |
| 10 | 66 " | 20 | 98 3-5° | Slight violet tint. | |
| 30 | 57 " | 24 | 98¼° | | Knees, ankles and elbows cool to touch and to the sense. Skin moist all over. Head feels light. |
| Hours. | | | | | |
| 3 | 60 | 22 | 98 1-5° | | A sensation of heat all over body; face flushed; feels exhilarated; head feels full. |
| 8 | 60 | 20 | 98¼° | Purple color. | |
| 12 | 60 | 18 | 98¼° | | Joints still cold. |
| Days. | | | | | |
| 2½ | 60 | 16 | 98¼° | Wine color. | No phenomenon present. |

It will be noticed as the result of these experiments, that the acid is rapidly absorbed, and in from four to ten minutes begins to be eliminated by the kidneys. The amount eliminated gradually increases during the first one or two hours, when the capacity of the kidneys seems to be reached, and the quantity eliminated remains quite constant during the next three to eight or more hours, according to the amount taken, and then it diminishes until it is all excreted, some two or three days after a single dose is taken; depending upon the amount taken.

The increase of pulse, respiration and temperature immediately after the taking of the acid is noticable in Experiment I. Then follows the rapid fall of the pulse to normal, and the respirations much below normal, while the temperature rises; but later the temperature also falls below normal, and then with the pulse and respiration it rises above again; and finally all return to the normal standard at the end of two and a half hours after taking the acid. But the remarkable effect, both in health and in disease, is the great reduction of the sensible and actual temperature of the extremities, and this reduction of temperature will be increased by larger or continued doses till the axillary temperature is decidedly lowered; a result which is especially noticed in disease. The acid seems to act especially upon the trophic nerve centres, if such centers exist, which preside over nutrition and the production of animal heat; lessening their activity, and thereby diminishing the temperature, especially in the extremities.

CHICAGO, 1108 Indiana Avenue.

